# Bioinspired Rigid–Flexible Coupled Adaptive Compliant Motion Control of Robot Gecko for Space Stations

**DOI:** 10.3390/biomimetics8050415

**Published:** 2023-09-06

**Authors:** Xiangli Pei, Shuhao Liu, Anmin Wei, Ruizhuo Shi, Zhendong Dai

**Affiliations:** 1 College of Mechanical and Electrical Engineering, Nanjing University of Aeronautics and Astronautics, Nanjing 210016, China; pxl_hebut@163.com (X.P.); 309770078@nuaa.edu.cn (S.L.);; 2 Institute of Bio-Inspired Structure and Surface Engineering, Nanjing University of Aeronautics and Astronautics, Nanjing 210016, China

**Keywords:** biomimetic robot gecko, adaptive compliance control, microgravity environment

## Abstract

This paper presents a study on bioinspired rigid-flexible coupling adaptive compliant motion control of a robot gecko with hybrid actuation for space stations. The biomimetic robot gecko is made of a rigid trunk, four motor-driven active legs with dual-degree-of-freedom shoulder joints, and four pneumatic flexible pleated active attachment–detachment feet. The adaptive impedance model consists of four input parameters: the inertia coefficient, stiffness coefficient, damping coefficient, and segmented expected plantar force. The robot gecko is equipped with four force sensors mounted on its four feet, from which the normal force of each foot can be sensed in real-time. Based on the sensor signal, the variable stiffness characteristics of the feet in different states are analyzed. Furthermore, an adaptive active compliance control strategy with whole-body rigidity–flexibility-force feedback coupling is proposed for the robot gecko. Four sets of experiments are presented, including open-loop motion control, static anti-interference experiment, segmented variable stiffness experiment, and adaptative compliant motion control, both in a microgravity environment. The experiment results indicated that the presented control strategy worked well and the robot gecko demonstrates the capability of stable attachment and compliant detachment, thereby normal impact and microgravity instability are avoided. It achieves position tracking and force tracking while exhibiting strong robustness for external disturbances.

## 1. Introduction

With the advent of the human space exploration fervor and the completion of space station construction, the demand for on-orbit services related to the safe and reliable operation of space stations has become increasingly pressing. The substance of on-orbit tasks has become more defined, diverse, and complex [1,2]. However, the harsh and complex environment of space, characterized by microgravity, intense radiation, and extreme temperature variations, poses great challenges for space transportation systems and astronauts alike. Any space vehicle or equipment is susceptible to failures or degradation at any time. Therefore, an increasing number of tasks require on-orbit servicing [1,2,3], such as space assembly, routine maintenance, extravehicular inspections, refueling, as well as the removal of orbital debris for satellites, spacecraft, and space stations, etc. Accomplishing these tasks solely with the assistance of astronauts entails high costs and risks. Consequently, it is imperative to develop a robot capable of stable crawling on confined surfaces in the microgravity environment of the space station. This would enable the automation of on-orbit operations, significantly reducing spacecraft costs, minimizing risks to astronauts, and enhancing operational capabilities in space [3]. However, the unique microgravity environment of space poses a significant challenge for achieving stable and reliable contact between mobile robots and spacecraft, which remains a major hurdle in the industry. Furthermore, the complexity of various spacecraft and space stations continues to escalate with the advancements in the space industry, imposing higher performance requirements on microgravity robots. Fortunately, after 3.5 billion years of evolution, animals have developed remarkable adhesion and locomotion mechanisms, which provide valuable insights for the study of biomimetic climbing robots and their applications in microgravity environments [3,4].

Over millions of years of evolution, geckos have possessed excellent climbing capabilities, such as highly efficient propulsion, high climbing speed, and strong adhesive forces, etc. In 2015, Russian scientists conducted relevant experiments on geckos in the microgravity environment of space. They observed geckos in space through the “BION-M” unmanned spacecraft and found that geckos could still maintain stable adhesion and complete pre-designed climbing tasks in a microgravity environment [5,6]. Inspired by geckos, engineers and scientists around the world have focused on the research of gecko-inspired robots, aiming to solve the challenge of stable attachment in microgravity through adhesion-based climbing. In 1966, researchers in Miyazaki University designed the first climbing robot, called Mod-1, which could move on vertical walls and ceilings like a gecko. Subsequently, in 1975, they completed the construction of the second-generation prototype, Mod-2 [7,8]. Since then, many different gecko-inspired robots have been designed and built [9,10,11]. From the perspective of mobility mechanisms in existing research, gecko-inspired robots can be categorized into four types: legged, wheeled, tracked, and hybrid mobility systems [9,10]. The wheeled design usually has simple structures, fast movement speeds, and relatively easy control, but it has weak obstacle-surmounting abilities and is suitable for relatively flat-structured surfaces. The tracked design effectively increases the contact area with the surface and enables continuous movement. However, it often has bulky structure, making turning challenging, and is more suitable for continuous-structured surfaces. The legged design can effectively simulate the locomotion of a gecko. It exhibits improved terrain adaptability and obstacle-surmounting capabilities compared with a wheeled and tracked design. It can be used on irregular and discontinuous unstructured surfaces. Admittedly, this design introduces complexity in synchronizing the control between legs, which presents challenges in the motion control of gecko-inspired robots. Examples of typical legged climbing robot prototypes include Stickybot [12], Abigaille [13,14], CLASH [15], Spinybot [16], RiSE [17], and SCALER [18]. However, they mostly work in gravity environments.

Additionally, various attachment methods, such as magnetic adhesion [19,20], pressure difference adsorption [21], claw spike attachment [16,17,18], and electrostatic adhesion [22,23] have been utilized in the development of gecko-inspired robots. Through proper control, these methods can achieve surface attachment. However, they are prone to limitations imposed by surface materials and have the potential to damage climbing surfaces. In contrast, adhesive-based bionic attachment methods do not damage climbing surfaces and can be applied to most relatively smooth spacecraft surfaces. The method has been proven to have high adhesive forces and are easy to implement [12,13,14,15]. Meanwhile, the challenge of achieving a balance between strong adhesion and robot maneuverability remains a difficult task in the motion control of bioinspired robots.

It is noted that in the early stages of research on gecko-inspired robots, the focuses were mainly on developing mechanical prototypes and bio-inspired adhesive materials, with limited reports on control methods. The control of gecko-inspired robots was typically done by open-loop position control strategies [12,15,17,24]. The next stage of robot gecko research requires the robot being able to perceive its surrounding environment and adapt it accordingly. In this case, feedback-based closed-loop control becomes essential. Furthermore, gecko-inspired robots in a microgravity environment face challenges such as instability, significant normal impacts, and poor environmental adaptability. However, a systematic design for closed-loop control that incorporates active control methods for motion, attachment, and detachment in a microgravity environment is still missing.

Our team has been working on bioinspired robots for many years [25,26,27,28]. Arthicha et al. designed a tailless gecko-inspired robot with flexible limbs and adhesive footpads that effectively mimics the natural climbing motion of geckos [26]. In 2021, Wang et al. proposed a simple online impedance strategy to control the peeling angle of the robot footpad for achieving compliant motion. The strategy was validated using the gecko-inspired robot IBSS-8. This impedance controller significantly reduced the sudden changes in normal adhesion force during the peeling process, enabling smooth detachment at a peeling angle of π [27].

The objective of this paper is to develop adaptative variable stiffness active compliant control for stable attachment and compliant detachment of the hybrid pneumatic–electric-driven system. The rest of the paper is organized as follows. Section 2 presents the biomimetic mechanism of the robot gecko. The design of the robot gecko is presented in Section 3. Section 4 presents the adaptative compliant control system. Section 5 presents the experimental results. Finally, Section 6 contains conclusions and future research topics.

## 2. Biomimetic Mechanism

**Figure 1:**
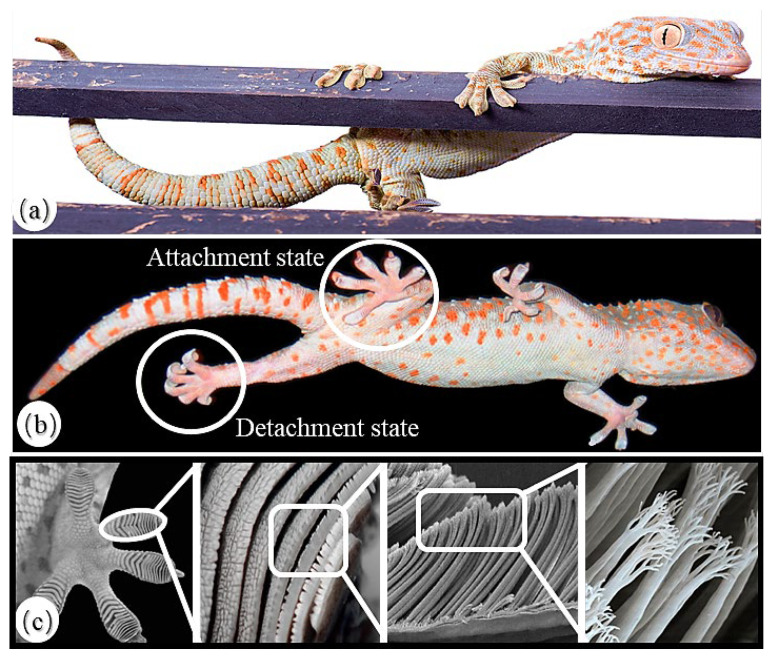
Morphological features of a gecko: (**a**) the climbing state of a gecko on a wooden pole; (**b**) the attachment state and detachment state of gecko feet [33]; (**c**) The microscopic structure of gecko feet [30].

The ventral side of each toe of the gecko is characterized by a series of arc-shaped folds, which are formed by proteinaceous setae measuring approximately 100 μm in length and 5 μm in diameter. These setae, numbering around 5000 per square millimeter, further split into spatula-shaped nanofibers at their tips, which can create a cup-like adhesive structure that maximizes the contact area [29,30]. Thus, a hierarchical and finely divided adhesive system is formed for the gecko. Figure 1 shows the foot structure of a gecko. The remarkable adhesive capability of geckos allows them to exhibit an exceptional climbing performance in various environments, which is attributed to the van der Waals forces generated by the countless setae structures [31]. The adhesion structure of a gecko’s foot has been confirmed to possess unique advantages, including the mechanisms of van der Waals forces, anisotropy, strong attachment, controllable detachment, anti-adhesiveness to itself, and self-cleaning properties [29,30,31,32]. By referring to the adhesion structure, it can be used in the design of a robot attachment system.

Geckos primarily inhabit crevices between rocks, caves, or tree cavities in jungles, deserts, rocky terrains, or wilderness areas. Occasionally, they can also be found near the eaves and walls of human dwellings. Geckos can navigate freely in such environments, which is mainly attributed to their flexible limbs, which enable them to adapt to the diverse and complex terrains found in nature, as well as their obstacle-crossing and wall-transition capabilities. By drawing inspiration from the locomotion of gecko limbs, it can be applied in the design of robotic locomotion mechanism.

Additionally, studies have revealed that distinct kinematic patterns and adaptive strategies are employed by a gecko during climbing on positive, zero, and negative surfaces [34]. By controlling the foot orientation, support angle, and locking mode, geckos adjust their attachment behavior to accommodate different angles’ surfaces [34]. They also adjust their locomotion behavior by regulating parameters such as center of mass velocity, stride length, stride frequency, and duty factor to adapt to different environments. Attachment and locomotion behaviors work together to enable successful climbing [34]. By referring to the coordination behavior and kinematic patterns of a gecko, it can be used in the design of a robot motion control system.

## 3. The Design of the Robot Gecko

### 3.1. Bionic Mechanical Design

**Figure 2:**
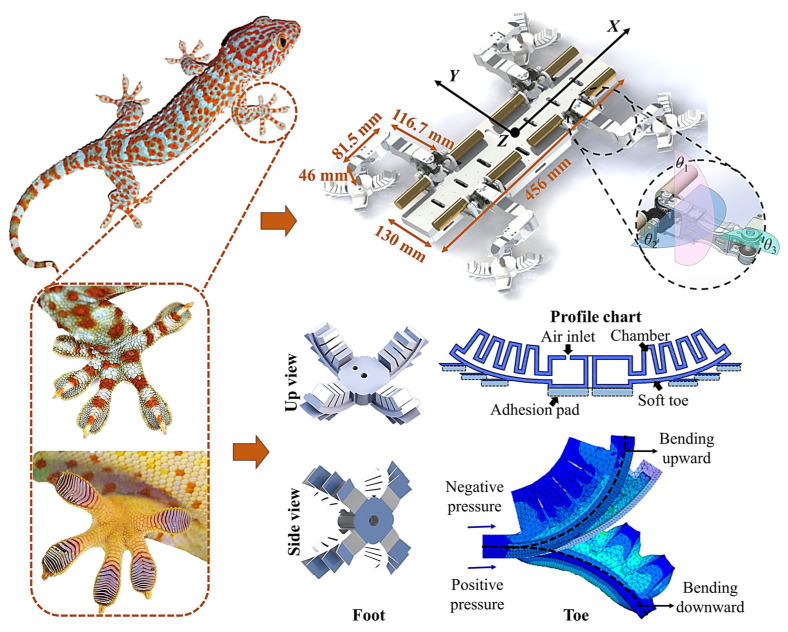
Biomimetic schematic of a gecko robot and its feet.

We develop a biomimetic hybrid pneumatic–electric-driven robot gecko that mimics the behavior of a real gecko. The structure and bionic concept of the robot gecko are depicted in Figure 2. The robot gecko comprises three parts, namely, a rigid torso, four electric-driven active limbs with dual-degree-of-freedom shoulder joints, and four pneumatic flexible pleated active attachment–detachment feet. It is 456 mm long, 402 mm wide (including the thighs and feet), and 84 mm high. The rigid torso serves as a platform for mounting and securing electronic components, such as the motor drive unit, pneumatic unit, and battery. The robot’s limbs are designed to mimic the crawling posture and knee-elbow in the opposite direction of quadrupedal reptiles like geckos. Each leg has three active degrees of freedom, with two servo motors providing roll and pitch motions for the thigh joint through a differential gear mechanism. This design enables the distribution of leg-lifting and leg-swinging torques between the two servo mechanisms. The pitch motion of the lower leg joint is controlled by a servo motor embedded within the thigh. Overall, the robot has six active degrees of freedom to control its center of mass. The front and hind limbs, as well as the left and right limbs, feature symmetric structures, enabling flexible movement in three-dimensional space. The force sensors are incorporated as a unified part for the lower legs. This design follows the principles of miniaturization and integration, ensures a coordinated structure of the robot, and minimizes the inertial interference caused by large rotational inertia at the foot.

The robotic system incorporates a pneumatic flexible wrinkled biomimetic foot, which aims to mimic the flexible hierarchical structure and adhesive characteristics observed in a gecko foot. As depicted in Figure 2, the foot design includes four pleated toes, with the undersides of the toes solidified with adhesive materials called a mushroom-shaped micro-structured adhesive [35]. Research has shown that the adhesive materials possess a certain level of adaptability to the space environment [36,37,38]. These toes are constructed as hollow structures with a strong internal seal. The driving mode of the foot is optional and subject to experimentation. For the purpose of simplicity and reproducibility, during the laboratory verification stage, we have chosen a pneumatic system as the driving mode for the flexible foot. When inflating (positive pressure) the foot, the toes expand outwards, enabling them to attach to the climbing surface. Conversely, when deflating (negative pressure) the foot, the toes curl upwards, facilitating detachment. In the inoperative state, the toes return to their natural position, that is, atmospheric pressure. For detailed design and analysis of the biomimetic foot, please refer to the given reference [39]. It shall be mentioned that our team members are constructing a liquid-driven system using ionic liquids as the fluid medium to meet the vacuum and extreme temperature conditions of space stations.

### 3.2. Biomimetic Hybrid-Driven Design

**Figure 3:**
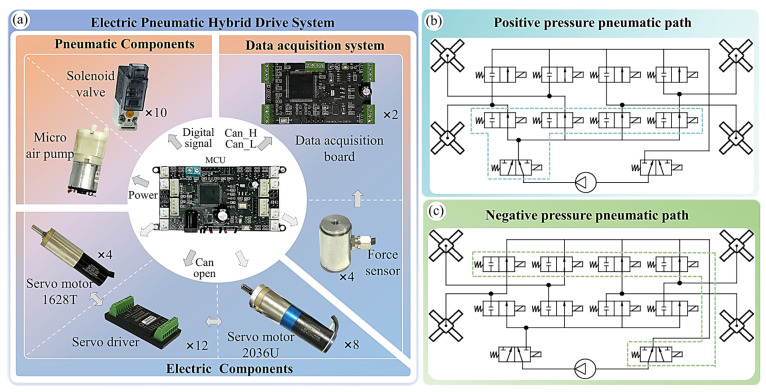
Electric pneumatic hybrid drive system of a robot gecko: (**a**) the hardware of the gecko robot; (**b**) schematic diagram of the positive pressure pneumatic path; (**c**) schematic diagram of the negative pressure pneumatic path.

The thigh and lower leg joints of a robot gecko are actuated by brushless DC servo motors; they operate in an electric-driven mode, while the attached feet utilize a pneumatic-driven mode. Therefore, the robot gecko is a hybrid drive system, which makes it become a more lifelike organism that is closer to a real gecko. The electronic devices used in the robot gecko are presented in Figure 3a. They mainly include the microcontroller unit (MCU), data acquisition board, pneumatic-driven components, electrical-driven components, sensors, and more. Using a 6S1P 2600 mAh polymer lithium battery as the power supply, it supports the robot for nearly 1 h of nonstop crawling according to our test. The STM32H743 microcontroller processes data and produces instructions. The FAULHABER 2036U 024B and FAULHABER 1628T 024B servo devices (Dr. Fritz Faulhaber GmbH & Co. KG; Stuttgart, German) are selected as the thigh and lower leg joints, respectively. The Dayang DYZ-102 normal force sensor (Bengbu ocean sensing system engineering Co., Ltd.; Bengbu, China) is used to dynamically measure the contact forces between the robot and its environment, enabling a real-time force feedback control strategy for the robot.

The pneumatic system is responsible for controlling the inflation and deflation of the foot. The air pathway must remain unobstructed to prevent blockages. For the air supply, the ASLONG PYP528-XA (AOLONG (HK) TECHNOLOGY Co., Ltd.; Shenzhen, China), a miniature DC silent vacuum pump, is selected, with a maximum positive pressure of 100 kPa. The pump is powered through the power module on the MCU. To control the airflow between the pump and the feet, two T-103-HM3/2 NC two-position, three-way solenoid valves are utilized as the main valves. The other eight T-103-HM2/2 NC two-position, two-way solenoid valves are selected as the unidirectional valves for the four feet. These valves allow for the switching of airflow direction, enabling inflation and deflation of the feet. These solenoid valves are securely installed on well-sealed valve seats. The four inlet/outlet unidirectional valve seats are interconnected, while the two main valve seats are disconnected. The air pathway diagram is shown in Figure 3b,c.

### 3.3. Design of Data Acquisition and Control System

The control system serves as the brain of the robot gecko, responsible for upper-level decision-making to ensure real-time performance and stability during its motion. In order to achieve synchronized control over various components, a high-performance, low-power, and miniaturized printed circuit board (PCB) is designed. The control chip employed is the STM32H743VIT6 with an ARM Cortex-M7 32-bit RISC core; with its frequency as high as 480 MHz, this chip facilitates efficient computation. Additionally, the PCB integrates the power module, communication module, and solenoid valve driver module, and more. The power module utilizes XN-50H and AMS1117 voltage-stabilizing chips, and provides a stable DC power supply of 3.3 V, 5 V, and 6 V for all the devices of the robot. To ensure reliable signal transmission with high real-time performance, flexibility, and anti-interference capability, the TJA1050 chip has been selected for the communication module. It is used for communication with motors and the data acquisition system. In order to relieve the burden on the main control chip, all the solenoid valves are driven by the S8050 module, which is integrated into the PCB. The reliability and convenience of physical connections are also considered; the servo devices and the pneumatic interfaces for the four feet are properly positioned at the four corners of the PCB, ensuring an optimal layout.

The data acquisition system is used for real-time and high-speed collection of analog signals from the force sensors on the robot’s feet. These signals are converted to digital signals and transmitted reliably to the control system to assist in decision-making. To provide scalability and portability for future use of multi-dimensional force sensors, an independent data acquisition system is designed. It utilizes the STM32F407 as the central computing unit. The system incorporates two 24-bit HX711 ADC chips to amplify and convert the weak mV signals from the sensors. The processed signals are then transmitted in real-time to the control system through the TJA1050 module. The flexibility and interchangeability of the signals are also considered; a single acquisition board that captures signals with two channels is designed. As a result, the data acquisition system consists of two acquisition boards, acquiring data from four sensors.

## 4. Adaptive Active Compliant Control Strategy

The contradiction between attachment and maneuverability poses a major challenge in the motion control of the robot gecko. Reliable attachment often implies difficulty in detachment; thus, an excellent motion control strategy that cannot only enhance the attached capability for stable attachment and enable agile locomotion through gait planning but also ensure smooth detachment without impact during the attachment-to-detachment transition of its feet is demanded. This presents another formidable problem for motion control.

The perspectives of locomotion behaviors and attachment behaviors are considered to solve the above issues. In terms of locomotion behaviors, for the foot trajectory planning, zero velocity and zero acceleration at the initial and final moments are considered. For the gait planning, a fulcrum-swinging gait (FSG) inspired by team biology experiments is proposed [40]. This gait ensures stable support from the hind legs and smooth movement of the forelegs. Regarding attachment behavior, considering that the uncertainty and randomness of the adhesion force make it difficult to establish a relatively accurate dynamic model of the robotic gecko, we instead focus on the variable stiffness characteristics of the robotic gecko and design the control law of the robotic gecko based on an impedance model. High stiffness control should be avoided during the attachment and detachment instants. During the attach phase, attachment force tracking with high compliance is achieved through admittance control. Based on the analysis of variable stiffness characteristics, an adaptive active compliant control strategy is employed for supporting and swinging legs. Furthermore, the coordination between the attachment–detachment mechanism and the limbs is considered in the control strategy to maximize attachment strength and minimize detachment impact.

### 4.1. Motion Planning Based on Bionic Inspiration

Motion planning is the key to the locomotion of the robot gecko. This mainly includes trajectory planning and gait planning.

#### 4.1.1. Centroid-Foot Coupling Motion Planning

The closed-chain multi-degree-of-freedom centroid-foot coupling motion planning model is established for the bioinspired robot gecko. The relative transformation between the robot gecko and the environment is described using the homogeneous transformation method. Firstly, four coordinate systems are established: the world coordinate system {*O_W_*}, the centroid coordinate system {*O_B_*}, the shoulder joint coordinate system {*O_S_*}, and the foot coordinate system {*O_F_*}. These coordinate systems form a closed-chain relationship as follows:

(1) $$T_{W}^{F}=T_{W}^{B}T_{B}^{S}T_{S}^{F}$$

where $T_{W}^{B}$ represents the transformation of the world coordinate system to the centroid. Similarly, $T_{B}^{S}$ represents the transformation matrix from the centroid to the shoulder joint. $T_{S}^{F}$ represents the transformation matrix from the shoulder joint to the foot. $T_{W}^{F}$ represents the transformation matrix from the world coordinate system to the foot.

The motion planning of the centroid is designed as a function of time. To simplify, the forward displacement of the centroid, *p_xwb_*, is generally planned as a linear function of time *vt* to account for a uniform forward motion. The lateral displacement, *p_ywb_*, is typically planned as zero during crawling experiments but can be planned as a linear function of time *vt* during turns. The vertical displacement, *p_zwb_*, is used to plan the height of the centroid and can be planned based on biomimetic principles, such as a certain proportion of the body length. If the centroid needs to fluctuate up and down, it can be planned as a linear function of time *vt*. The roll angle, *R*, is generally planned as zero. The pitch angle, *P*, can be set as a constant value corresponding to different preloads of the front and hind legs. The yaw angle, *Y*, can be planned as a linear function of time ωt during robot turns or biomimetic swing.

For the convenience of model establishment and subsequent computation, the centroid-foot coupling motion planning method is adopted in this paper. The trajectory of the foot’s center point is planned relative to the world coordinate system. That is completed in matrix $T_{W}^{F}$. It is represented as a function of spatial position with respect to time. The trajectory planning of the foot is divided into four segments according to the motion of the foot; hereinafter, the four segments are referred to as force control phases in this paper:

1. Attachment segment (AS): The foot finds the landing point and inflates the toes of the foot in response to environmental force feedback. Thus, the attachment process is completed. During this stage, the position of the foot relative to the world coordinate system remains unchanged, relying mainly on the characteristics of the foot for attachment.
2. Support segment (SUS): The foot maintains the attachment state. During this stage, the position of the foot remains unchanged.
3. Detachment segment (DS): When detachment is required, the foot deflates the toes of the foot to complete detachment. During this stage, the position of foot remains unchanged, relying on the characteristics of the foot, just the same as the attachment segment.
4. Swing segment (SWS): After detachment, the foot enters the swing phase, and the single leg takes a step forward. During this stage, the position of the foot changes, and planning in three directions individually is required.

Therefore, the trajectory of the foot in attachment–support–detachment segments is planned as constants to meet the requirements. The trajectory design of the swing segment should consider the following factors: (1) a certain preload space for the attach process to generate sufficient attachment force. (2) Smooth and coordinated movement without significant up and down fluctuations, left–right tilting, or front–back shocks. (3) Smooth trajectory with zero velocity and acceleration at the start instant and end instant of the step to achieve zero-impact leg lifting and soft landing. (4) Parameter selection based on biomimetic inspiration.

The trajectories of the front and hind legs are separately planned according to their respective characteristics and requirements. The equation of the improved composite cycloidal trajectory planned for front legs is as:

(2) $$\left\{\begin{array}{l} x=S_{roboti}\left[t/T_{swing}-\sin\left(2\pi t/T_{swing}\right)/2\pi \right]\\ z=H_{robot}\left[\mathrm{sgn}\left(T_{swing}/2-t\right)\left(2f_{E}\left(t\right)-1\right)+1\right] \end{array}\right.$$

where

$$\begin{array}{l} f_{E}\left(t\right)=t/T_{swing}-\sin\left(4\pi t/T_{swing}\right)/4\pi \\ \mathrm{sgn}\left(T_{swing}/2-t\right)=\left\{\begin{array}{l} 1\;\;\;0\leq t<T_{swing}/2\\ -1\;T_{swing}/2\leq t<T_{swing} \end{array}\right. \end{array}$$

**Figure 4:**
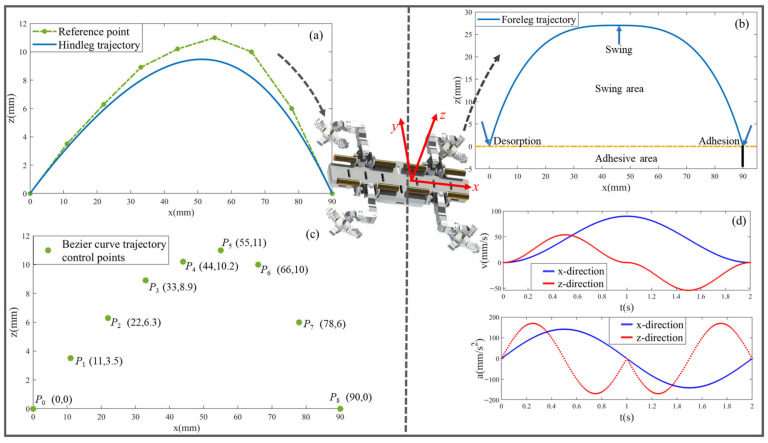
The trajectory planning results of the front and hind legs of the robot gecko: (**a**) the hindleg trajectory; (**b**) the foreleg trajectory; (**c**) Bezier curve trajectory control points; (**d**) speed and acceleration of the front legs.

The equation of the biomimetic 8th-order Bezier curve trajectory planned for the hind leg is as:

(3) $$\begin{array}{l} B(u)={\left(1-u\right)}^{8}p_{0}+8u{\left(1-u\right)}^{7}p_{1}+28u^{2}{\left(1-u\right)}^{6}p_{2}+56u^{3}{\left(1-u\right)}^{5}p_{3}+70u^{4}{\left(1-u\right)}^{4}p_{4}+\\ 56u^{5}{\left(1-u\right)}^{3}p_{5}+28u^{6}{\left(1-u\right)}^{2}p_{6}+8u^{7}\left(1-u\right)p_{7}+u^{8}p_{8} \end{array}$$

where *p*_0_ to *p*_8_ are control points, with coordinates as shown in Figure 4c. The parameter u is normalized by a minimum unit of 0.01.

The trajectory planning curves for the front and hind legs are illustrated in Figure 4a,b, respectively. In Figure 4d, the velocity and acceleration of the foreleg is demonstrated. It can be observed that the velocity and acceleration transition smoothly without any aberrations at specific positions, and stable leg stepping is guaranteed. After landing, a certain space of vertical downward preload is provided to facilitate the generation of attachment force. In Figure 4a, it is evident that the front segment of the swing is gentle, while the subsequent segment becomes steeper. As the fulcrum, the hind leg must always guarantee its own stability.

#### 4.1.2. Biomimetic Gait Design for Microgravity

In this paper, there are three kinds of gait used for robot gecko movement in the microgravity environment: triangular gait (TG), diagonal gait (DG), and fulcrum-swing gait (FSG). The duty cycle for the triangular and diagonal gaits are 0.75 and 0.5, respectively, which have been relatively matured and will not be further elaborated here. The fulcrum-swing gait, situated between the two, is an improved biomimetic gait suitable for aerospace microgravity environments.

**Figure 5:**
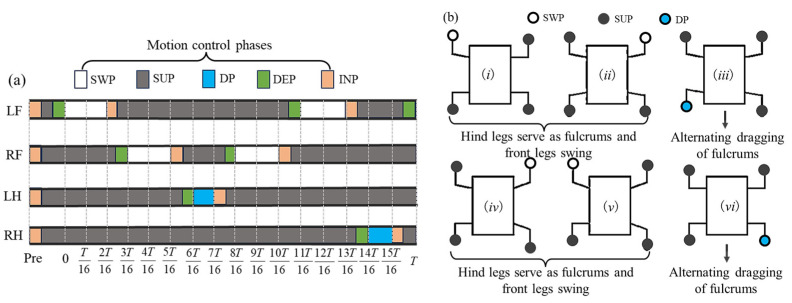
The alternate fulcrum-swing gait: (**a**) the phase diagram; (**b**) the schematic diagram (*i*–*vi* indicates the step sequence).

**Figure 6:**
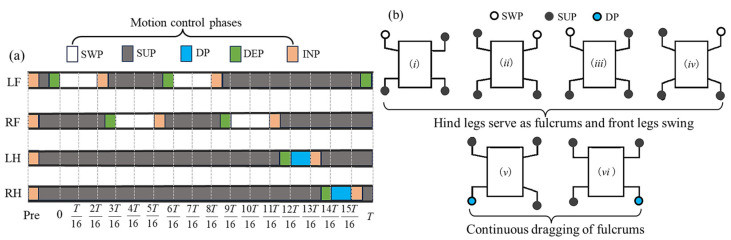
The continuous fulcrum-swing gait: (**a**) the phase diagram; (**b**) the schematic diagram (*i*–*vi* indicates the step sequence).

In the fulcrum-swing gait, priority is given to the alternating swing of the front legs, while the hind legs act as the fulcrum for support. The torso swings around the fulcrum along with the front legs and centroid. Within one gait cycle, the front legs swing twice alternately, while the hind legs swing once. Therefore, the number of steps with the front legs is greater than that of the hind legs. The single stride distance of the front legs should be smaller than that of the hind legs, and the step height of the front legs is greater than that of the hind legs. The front legs adopt the improved composite cycloidal trajectory, while the hind legs follow the Bezier curve trajectory. The centroid can have a slight positive pitch angle to provide additional downward pressure for the hind legs and increase stability. To ensure stability, there should be a certain period of four-legged support between any two consecutive swings within one gait cycle. During this period, only the centroid moves forward. The entire gait cycle satisfies the following: the hind legs provide stable support, and the front legs provide propulsion. Inspired by geckos, two types of FSG are designed in this paper: alternate fulcrum-swing gait (A-FS) in Figure 5 and continuous fulcrum-swing gait (C-FS) in Figure 6.

**Remark 1**. *The usage and explanation of SWP, SUP, DP, INP, and DEP are as follows: SWP: swing phase, SUP: support phase, DP: drag phase, INP: inflation phase, DEP: deflation phase; these five belong to the motion control phase in this paper*.

**Remark 2**. *For simplicity, the LF, RF, LH, RH are used to represent the left front leg, right front leg, left hind leg, and right hind leg of the robot gecko in this paper*.

### 4.2. Adaptive Variable Stiffness Active Compliance Control

**Figure 7:**
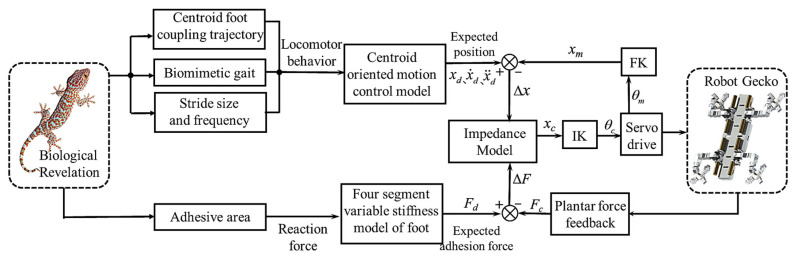
The control structure of the robot gecko.

In this section, we present an adaptive variable stiffness active compliance control system with rigid–flexible–force feedback fusion for the hybrid-driven robot gecko, as depicted in Figure 7. Inspired by gecko locomotion, in terms of motion reaction forces, we generate the expected adhesion force *F_d_* of the robot gecko based on the trend of adhesive area variation of gecko feet and form a force deviation Δ*F* with the force sensor feedback *F_c_*. In terms of locomotor behavior, we obtain the expected motion parameters $x_{d}$, ${\dot{x}}_{d}$, ${\ddot{x}}_{d}$ through motion planning and form a position deviation Δ*x* with the motion parameters *x_m_* computed by forward kinematics (FK). The impedance parameters *M*, *B*, and *K* are derived through the segmented variable stiffness characteristics of the robot. Thus, the current motion parameters *x_c_* are exported by the impedance model, and the joint variables *θ_c_* are calculated by inverse kinematics (IK). In this way, the motion control of the robot gecko is accomplished. The proposed control strategy enables the robot to maintain stability and accuracy during interaction, while also providing compliance and adaptability to handle uncertainties and disturbances.

#### 4.2.1. Active Compliance Control

Generally, the mathematical relationship for an impedance model is established as follows:

(4) $$m\ddot{x}+b\dot{x}+kx=f$$

where *x* represents the position, *m* the mass, *b* the damping coefficient, *k* the stiffness coefficient, and *f* the applied external force. By applying the Laplace transform to Equation (4), we obtain:

(5) (ms+bs+k)X(s)=F(s)

The transfer function of the impedance control is: *Z*(*s*) = *F*(*s*)/*X*(*s*); the force is controlled based on the positional deviation. The transfer function of the admittance control is: Y(s) = *Z*^−1^(*s*) = *X*(*s*)/*F*(*s*); the position is controlled based on the force deviation. In this paper, the motor utilizes the Cyclic Set-Point (CSP) motion mode; thus, the admittance control is used to achieve active compliance.

The relationship between the position error of the robotic gecko’s foot and the contact force applied to the foot can be described by an impedance equation as follows:

(6) $$M({\ddot{x}}_{d}-{\ddot{x}}_{c})+B({\dot{x}}_{d}-{\dot{x}}_{c})+K(x_{d}-x_{c})=F_{d}-F_{e}$$

where *M*, *B*, and *K* are impedance parameters, representing the inertia coefficient, damping coefficient, and stiffness coefficient, respectively. $x_{d},{\dot{x}}_{d},{\ddot{x}}_{d}$ represent the desired values of the position, velocity, and acceleration, respectively. $x_{a},{\dot{x}}_{a},{\ddot{x}}_{a}$ represent the actual values of the position, velocity, and acceleration. *F_d_* represents the desired external force/torque, while *F_e_* denotes the actual external force/torque from the environment. Readers can understand this as an impedance-based system receives input from an admittance-based external system, resulting in complementary effects to counteract bidirectional deviations in position and force. The supplemental quantity of admittance is instantaneously fed back into the equation, and a dynamic balance between the impedance system and the admittance system is achieved.

Let $\Delta F=F_{d}-F_{e}$; Equation (6) implies:

(7) $${\ddot{x}}_{c}(i)={\ddot{x}}_{d}(i)-M^{-1}\{\Delta F(i)-B[{\dot{x}}_{d}(i)-{\dot{x}}_{c}(i-1)]-K[x_{d}(i)-x_{c}(i-1)]\}$$

where *i* represents the *i*_th_ computational cycle, and *i* − 1 represents the data from the previous cycle. Thus, the real-time acceleration ${\ddot{x}}_{c}\left(i\right)$ can be treated as a known quantity, and, through integration, ${\dot{x}}_{c}\left(i\right)$ and $x_{c}\left(i\right)$ can be obtained as follows:

(8) $$\left\{\begin{array}{l} {\dot{x}}_{c}(i)={\dot{x}}_{c}(i-1)+{\ddot{x}}_{c}(i)୵T\\ x_{c}(i)=x_{c}(i-1)+{\dot{x}}_{c}(i)\cdot T \end{array}\right.$$

where *T* represents the number of cycles for algorithmic computation. In each cycle, the value of $x_{c}\left(i\right)$ is calculated using Equation (8) and is then utilized as $x_{c}\left(i-1\right)$ in the next iteration. Then continuous positions are obtained to realize the real-time control of the robot. The controlled physical system exhibits the abilities of position and constant-force tracking. In the presence of external disturbances, the physical system can dynamically adjust in real-time to return to the desired physical state, demonstrating adaptability to the environment.

Considering that the robot gecko in this study possesses the ability to perceive the normal forces from its feet, an active compliant admittance control method of the feet is employed. Taking the right forelimb as an example, the desired normal position *z* is provided during motion planning in Section 4.1. Based on this, the final target position value $z^{\prime }$ is obtained by adding the increment Δz calculated by the admittance control algorithm to *z*, as follows:

(9) $$z^{\prime }=z+\Delta z$$

Regarding the desired force, let us assume that the single leg motion occurs within the null space. In this case, the desired normal force throughout the entire process is set to zero, i.e., in Equation (6), *F_d_* = 0. Meanwhile, *F_e_* represents the actual force acquired by the sensor. The compliance of the force feedback control can be expressed as follows:

(10) $$\Delta F=K_{G}\left(F_{d}-F_{e}\right)$$

where *K_G_* represents the gain value for force control.

**Figure 8:**
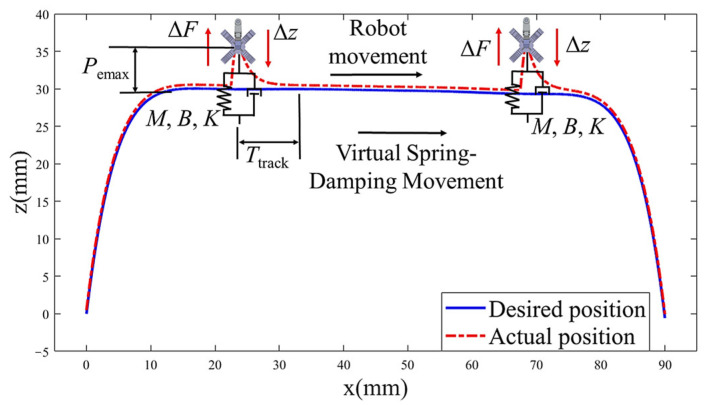
Virtual spring-damping schematic diagram.

The final actual position *X_c_* can be obtained from Equations (7), (8) and (10). The compliance of the position tracking can be expressed as follows:

(11) $$\Delta z=K_{M}X_{c}\left\{M,B,K\right\}$$

where *K_M_* represents the gain value for position tracking. Based on this, an active compliance relationship between ΔF and Δz is established, i.e., a real-time virtual “spring-damper” system is created between the desired position and actual position. The origin of the spring changes continuously with the desired position, which is an absolute reference frame, as shown in Figure 8. According to the Lyapunov’s second method, the impedance parameters *M*, *B*, and *K* of the virtual spring-damper system directly affect the stability of the mentioned control system. Therefore, appropriate impedance parameters should be chosen to ensure the stability of the system.

#### 4.2.2. Segmented Variable Stiffness Strategy

The compliance performance of the virtual spring-damper system of robot’s foot depends on the impedance parameters *M*, *B*, and *K*. By adjusting these parameters, the position tracking and force tracking characteristics of the robot gecko are analyzed, which can provide a basis for the segmented variable stiffness strategy of the foot.

**Figure 9:**
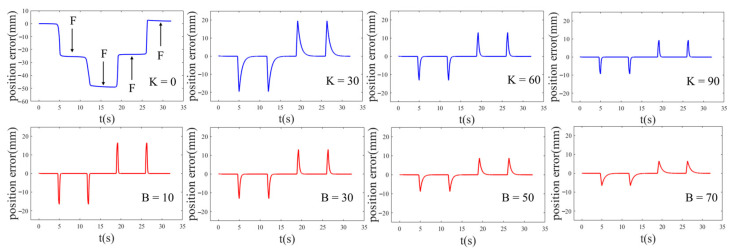
The influence of variable stiffness and damping on position tracking. The blue line represents the change of position error with K, and the red line represents the change of position error with B.

Taking the right forelimb as an example, it is made to continuously perform two swing-support cycles within the null space, and it follows the trajectory planned by Equation (2). In the first cycle, reverse disturbance forces are applied during the swing and support phases, while in the second cycle, forward disturbance forces are applied during the swing and support phases. The changes of position deviation are shown in Figure 9.

From Figure 9, the following conclusions can be drawn:

1. The actual position deviates due to external disturbing forces. After the disturbance is withdrawn, the actual position starts to follow the desired trajectory;
2. With M and B unchanged (*M* = 1, *B* = 20), when *K* = 0, the robot lacks stiffness and does not track the desired position. When *K* = 30, the position begins to follow, with an average position tracking time of 2.69 s. That is 1.75 s when *K* = 60, 0.61 s when *K* = 90. The data shows that under the same external disturbance, the position tracking time decreases with increasing stiffness. The stiffness coefficient affects the position tracking performance;
3. With M and K unchanged (*M* = 1, *K* = 30), when *B* = 10, the average position tracking time is 0.53 s. When *B* = 30, that is 1.02 s, 2.84 s when *B* = 50, and 3.88 s when *B* = 70. The data shows that under the same external disturbance, the position tracking time increases with increasing damping. The damping coefficient affects the response speed of position tracking;
4. Different stiffness and damping exhibit completely different force control characteristics and are suitable for different application scenarios. When flexible contact is required, stiffness is reduced, while stiffness is increased when disturbance rejection is needed. This reflects the variable stiffness characteristics of the robot motion.

Based on the above analysis, the four force control phases of AS-SUS-DS-SWS exhibit different contact and free space characteristics, thus necessitating the design of a segmented variable stiffness strategy. In DS: the smaller normal detachment force is expected, stiffness is reduced, and damping is increased to control the tangential detachment direction and avoid normal impact. In SWS: which corresponds to the free null space, position tracking is directly performed according to the foot trajectory planning results, with high stiffness to improve response speed and disturbance rejection. In AS: inspired by biomimetics, appropriate pre-pressure is applied to ensure stable attachment. The stiffness and desired attachment force should be minimized in the moment of attachment to prevent excessive reverse impact. In SUS: low stiffness and high damping are set to ensure a constant desired attachment force, enabling rapid and stable force tracking. Additionally, for the FSG, where the hind legs act as fulcrums to enhance stability and the front legs facilitate swinging and body movement by increasing step frequency, a variable stiffness strategy for the front and hind legs is designed to achieve stable attachment and compliant detachment, avoiding impact and instability problems while maintaining strong robustness.

It shall be mentioned that while the presented control strategy is derived for the robot gecko that has four electric-driven active limbs and four flexible feet, it is also applicable to other legged robots, such as a quadrupedal robot dog. In this case, the kinetic and dynamic models will be different, while the sensing can be the same. Due to the change of stiffness characteristics, the impedance parameters connected to the controller will be different.

## 5. Experiments

Several experiments are conducted to investigate the adaptive variable stiffness active compliant control design on a microgravity experimental platform. For satisfying various experimental requirements, a suspension method is employed to simulate a microgravity environment. A camera is installed across from the arc surface to shoot the robot gecko.

### 5.1. Construction of Microgravity Simulation Experimental Platform

**Figure 10:**
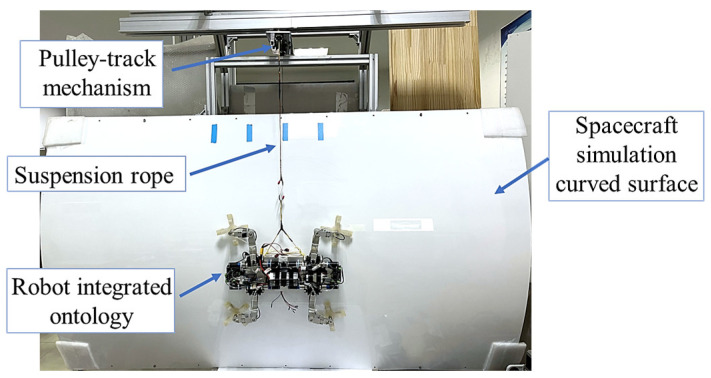
The suspended experimental platform simulating a microgravity environment.

A suspension method is utilized to simulate the microgravity environment for the robot gecko. This method relies on the vertical tension exerted by suspension wires to counterbalance the gravitational forces of the experimental object. The suspended microgravity platform is depicted in Figure 10. This platform consists of several components, including a pulley-track mechanism, a rope mechanism, counterweights, a simulated spacecraft surface, and a truss structure. By employing pulleys, the rope mechanism suspends the counterweight on one side, while the robot is tethered on the other side with a triangular structure. The pulley and track form a horizontal follow-up system. Serving as a proportional simulation of the spacecraft’s curved exterior surface, an acrylic surface is securely fastened beneath the track. The truss structure plays a crucial role in providing structural support and linking different modules together. This setup enables the robot to achieve horizontal climbing locomotion on the 90° (relative to ground) curved simulated surface.

### 5.2. Open-Loop Motion Control Experiment

The open-loop motion control experiments are conducted using the open-loop control without any feedback, where the desired joint positions obtained from motion planning are directly sent to the motors. The minimum computing unit is 10 ms. Four different gaits, i.e., TG, DG, C-FS, A-FS, are implemented, respectively. The foot force data of the four legs are recorded during the experiments. The experimental parameters are depicted in Table 1.

**Figure 11:**
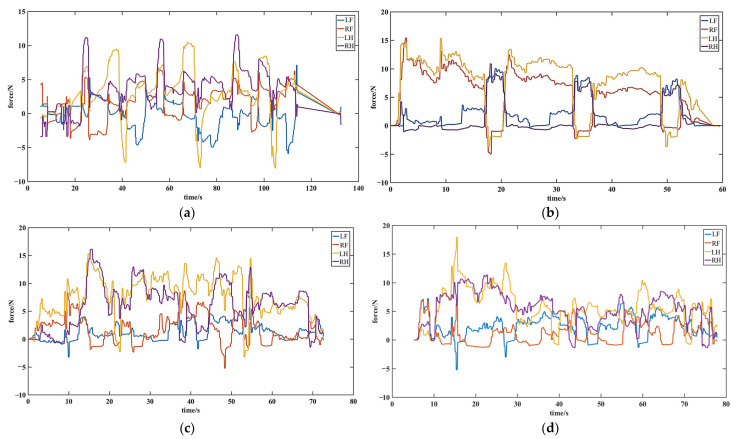
The force-time data of four gaits with open-loop motion control: (**a**) the force-time data of the triangle gait; (**b**) the force-time data of the diagonal gait; (**c**) the force-time data of the alternate fulcrum-swing gait; (**d**) the force-time data of the continuous fulcrum-swing gait.

Figure 11 presents the force-time data of four gaits. From the force-time data of the triangle gait in Figure 11a, it can be observed that the normal forces during the supporting phase are maintained within the range of 5 N to 10 N. Additionally, there are noticeable reverse impacts when the legs detached, indicating unstable attachment forces without any feedback control. From the force-time data of the diagonal gait in Figure 11b, it can be observed that the force variation trend of the two diagonal legs that move at the same time is similar. The normal forces during the supporting phase of each leg are maintained within the range of 5 N to 10 N. However, same as the triangular gait, the uncontrolled attachment forces remain unstable, thereby introducing destabilizing factors for disturbance rejection and crawling stability. As shown in Figure 11c, it is in the alternate fulcrum-swing gait that the normal forces of the two hind legs are larger than those of the two front legs. This indicates that the robot relies on its two hind legs for support, with longer support duration and only brief periods of dragging. As a result, this gait exhibits higher stability compared to the previous two gaits. However, the overall force data is not smooth, displaying significant fluctuations, and some leg movements exhibit notable impact forces during detachment. In Figure 11d, the changing trend of the continuous fulcrum-swing gait resembles that of the alternate fulcrum-swing gait. During the latter part of the cycle, the two hind legs continuously drag, which may result in momentary detachment of the fulcrum, thereby causing instability.

### 5.3. Static Anti-Interference Experiment

**Figure 12:**
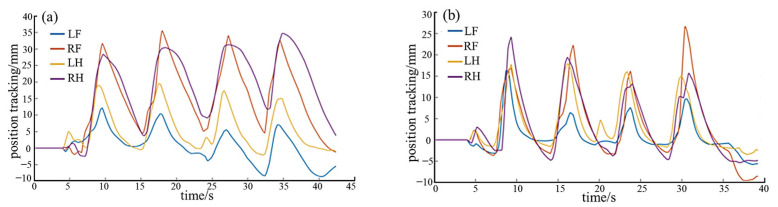

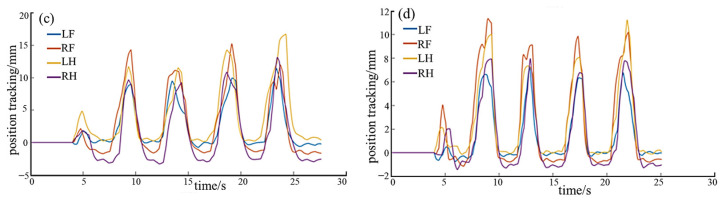
The position tracking with different stiffness: (**a**) K = 0; (**b**) K = 30; (**c**) K = 60; (**d**) K = 90.

**Figure 13:**
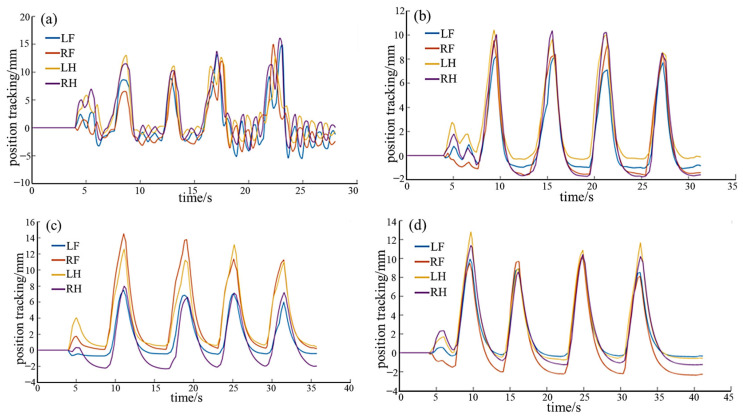
The position tracking with different damping: (**a**) B = 10; (**b**) B = 30; (**c**) B = 50; (**d**) B = 70.

Based on the analysis of variable stiffness characteristics of the robot gecko in the null-space (non-contact state) in Section 4.2.2, the whole-body static anti-interference experiments of the robot in the non-null space (environment-contact state) are conducted. The focus is on testing the anti-interference ability during the static attachment state, that is, the positional tracking performance. During the experiments, the robot is steadily attached to the surface under a microgravity environment. In each experiment, four normal interfering forces are separately applied and then revoked. It should be noted that the interference force remains the same for each experiment. To ensure consistency, the parameters *M*, *B*, *K*, are the same as those in Section 4.2.2. The position tracking results are shown in Figure 12 and Figure 13. The mean position deviations (MPD) and mean response (MRT) time are shown in Table 2 and Table 3.

As shown in Figure 12 and Table 2, under the same external force disturbance, the ability of position tracking varies with different *K*, while *M* and *B* held constant (*M* = 1, *B* = 20). The position deviation decreases with an increase in stiffness, while the position tracking time decreases as well. The experimental results are consistent with the analysis in Section 4.2.2. A smaller K enhances the compliance of the foot, allowing it to closely follow external forces and deviate from the desired position. However, in the experiment, the robot struggles to return to the desired position after being subjected to external forces, indicating high flexibility but limited resistance to disturbances. Conversely, a larger K results in higher stiffness. The robot promptly returns to the initial position after experiencing external forces. In the experiment, it demonstrates strong resistance and precise position tracking capabilities. The virtual spring quickly pulls the robot back to the initial position after any deviation, ensuring stable attached contact of the four legs without instability issues, showcasing excellent disturbance resistance.

From Figure 13 and Table 3, it is evident that under the same external force disturbance, with *M* and *K* kept constant (*M* = 1, *K* = 30), the position tracking performance is affected by the damping value. There is no significant change in position deviation, while the position tracking time increases with an increase in damping. Therefore, when stiffness remains constant, the damping value affects the response speed. The conclusions are same as the analysis in Section 4.2.2. A smaller *B* value leads to a faster response speed, although excessively low damping can result in oscillations. In the experiment, the robot quickly follows the desired position after the withdrawal of external forces. On the other hand, a larger *B* value corresponds to a slower response speed. In the experiment, the robot gradually follows the desired position after the withdrawal of external forces, maintaining stable attachment of the four legs without instability issues.

### 5.4. Segmented Variable Stiffness Experiments

**Figure 14:**
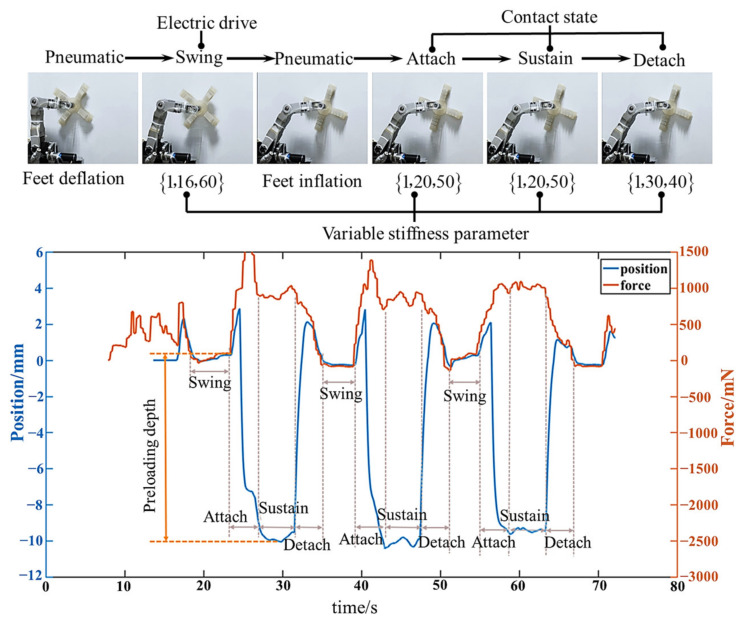
The analysis diagram of the experimental process when *F_d_* is 11 N.

The static anti-interference experiment in Section 5.3 is a verification of the variable stiffness characteristics of the robot. The single leg segmented variable stiffness experiments are conducted to demonstrate the practicability of the segmented variable stiffness strategy. During the experiment, the robot was vertically suspended on the simulated arc surface, ensuring that three legs, except for the LF leg, stably attached to the surface, providing support. The center of mass remained stationary. The LF leg executed reciprocating motion following the pre-designed trajectory in Section 4.1, that is, a cycle of swing–attachment–support–detachment. The segmented variable stiffness parameters {*M*,*B*,*K*} are determined according to the experimental results in Section 5.3, as shown in Figure 14.

The experimental process diagram and a data plot depicting the actual forces and preloading depths for an expected attachment force of 11 N are shown in Figure 14. It reveals that during the swing phase, the desired force on the foot is set to zero, resulting in zero position and force tracking. In the attachment phase, the robot initiates tracking of the desired attachment force, generating active preloading depths to increase the attachment force. The preloading depth exhibits a significant increase. During the support phase, the preloading depth is maintained to achieve constant force tracking. In the detachment phase, with the expected attachment force set to zero, the active preloading depth decreases. At the instant just before swinging, the actual force reduces to zero, successfully achieving smooth detachment without reverse impact.

**Figure 15:**
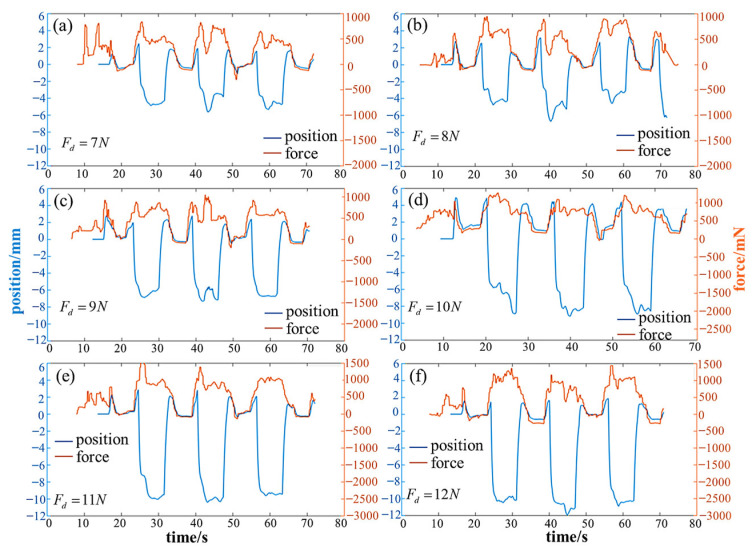
The actual forces and preloading depths when desired attachment forces *F_d_* are 7 N–12 N.

Likewise, the several other experiments are conducted with the same segmented variable stiffness parameters shown in Figure 14, while the desired attachment force varied from 7 N to 12 N. The obtained data of actual forces and preloading depths are presented in Figure 15 and Table 4. It can be observed that as the desired force increases, the active preloading depth and attachment force both increased, providing conditions for stable attachment. Meanwhile, the control strategy enables smooth detachment. The single leg segmented variable stiffness experiment lay the foundation for subsequent whole-body adaptative compliant motion control experiments.

### 5.5. Adaptive Compliant Motion Control Experiment

Several sets of whole-body adaptative compliant motion control climbing experiments are conducted for the four different gaits, i.e., TG, DG, C-FS, A-FS. The input parameters for motion control phase of each gait including *S*_robot_, *H*_robot_, *T*_gait_, *T*_in_, *T*_out_, are as described in Table 1. On this basis, the force control phase is added to one motion cycle. The input parameters for force control phase included desired plantar force *F_d_* and variable stiffness parameters {*M*,*B*,*K*} for each segment. In the climbing experiment, the robot successfully completed several gait cycles under the influence of the force control algorithm.

**Figure 16:**
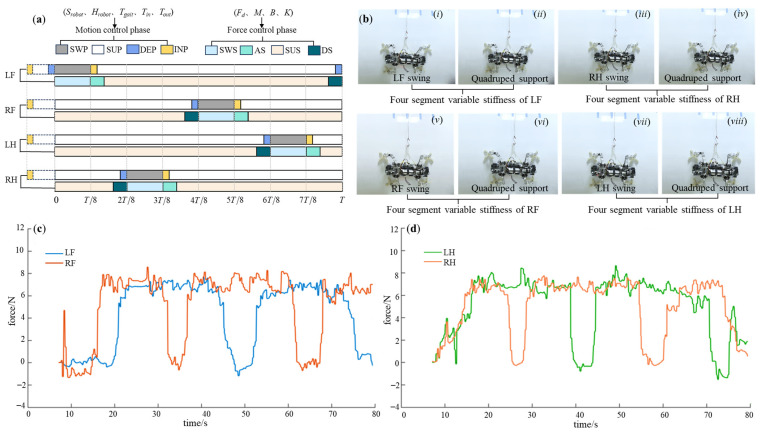
The experimental results of triangular gait. (**a**) The control phase diagram; (**b**) the experimental process (*i*–*viii* indicates the step sequence); (**c**) the force-time data of LF and RF; (**d**) the force-time data of LH and RH.

For the triangular gait, the force control parameters for each leg are shown in Table 5. The control phase diagram is depicted in Figure 16a. The experimental process is illustrated in Figure 16b, and the obtained plantar force data curves are shown in Figure 16c,d. It can be observed that both the front and hind legs closely follow the desired attachment force. Both legs act as propulsion without any apparent feet acting as support. Smooth detachment is achieved through variable stiffness, resulting in no impact. Ultimately, the triangular gait achieved a movement speed of 0.6 cm/s.

**Figure 17:**
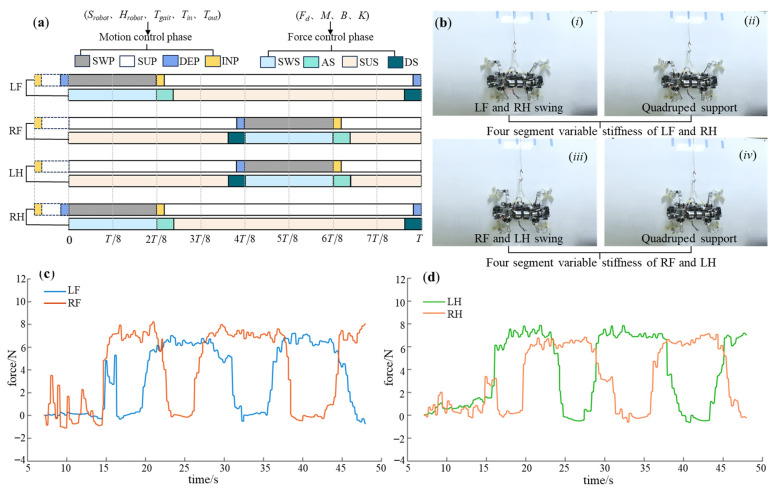
The experimental results of the diagonal gait. (**a**) The control phase diagram; (**b**) the experimental process (*i*–*iv* indicates the step sequence); (**c**) the force-time data of LF and RF; (**d**) the force-time data of LH and RH.

Similarly, for the diagonal gait, the force control parameters are shown in Table 5, and the control phase diagram is presented in Figure 17a. The experimental process is depicted in Figure 17b, and the obtained plantar force data curves are shown in Figure 17c,d. It can be observed that both the front and hind legs closely follow the desired attachment force. When detached, it exhibits flexibility through variable stiffness, resulting in an impact-free process. Ultimately, the diagonal gait achieved a movement speed of 1.2 cm/s; however, there is a certain degree of instability during double-foot support.

**Figure 18:**
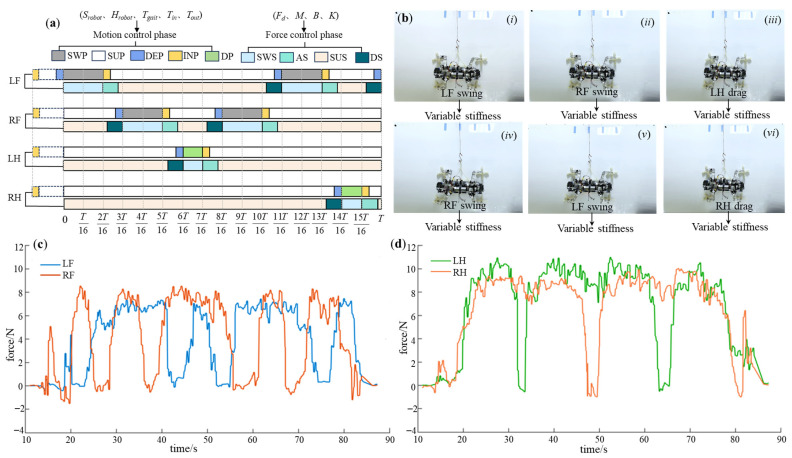
The experimental results of the A-FS gait. (**a**) The control phase diagram; (**b**) the experimental process (*i*–*vi* indicates the step sequence); (**c**) the force-time data of LF and RF; (**d**) the force-time data of LH and RH.

For the alternate fulcrum-swing gait, attention should be paid to the differences in force control parameters between the front and hind legs, ensuring that the desired attachment force of the hind leg is greater than that of the front leg, and the desired force of the left hind leg is greater than that of the right hind leg. In addition, the front legs have lower stiffness and higher damping than the hind legs, except for the swing segment. The specific force control parameters are presented in Table 6, and the control phase diagram is depicted in Figure 18a. The experimental process is illustrated in Figure 18b, and the obtained foot force data curves are shown in Figure 18c,d. It reveals that both front legs closely track the desired force during the attachment–support phase, while the hind legs demonstrate the same force tracking characteristics, with the actual force of the hind legs exceeding that of the front legs. Moreover, the actual force of the left hind leg is higher than that of the right hind leg. As support points during the support phase, the hind legs ensure stable attachment, and the front legs act as propulsion, achieving forward motion through a high stepping frequency. A movement speed of 0.44 cm/s is achieved at last. The detachment process smoothly follows until reaching zero through the virtual spring-damping system, resulting in zero impact in the moment of detachment. This meets the requirements for the support–swing attached behavior, and the robot exhibits excellent stability.

**Figure 19:**
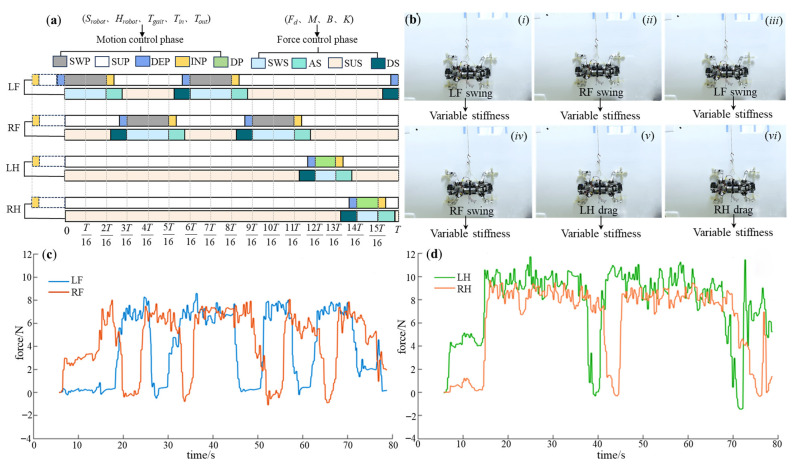
The experimental results of the C-FS gait. (**a**) The control phase diagram; (**b**) the experimental process (*i*–*vi* indicates the step sequence); (**c**) the force-time data of LF and RF; (**d**) the force-time data of LH and RH.

Likewise, for the continuous fulcrum-swing gait, the force control parameters are presented in Table 6, and the control phase diagram is depicted in Figure 19a. The experimental process is illustrated in Figure 19b, while the obtained foot force data curves are shown in Figure 19c,d. It can be observed that the front and hind legs of the C-FS demonstrate the similar force-tracking characteristics with the A-FS, satisfying the gait characteristics of the fulcrum-swing gait. The achieved motion speed is 0.44 cm/s. The detachment process can smoothly follow to zero with no detachment impact. However, during the continuous dragging of the two hind legs at the end of a gait cycle, opportune stable support cannot be provided, leading to instability of the robot during this interval. The plantar force exhibits impact and disorder in this period. Consequently, the C-FS is not as stable as A-FS.

## 6. Conclusions

This paper presents a bioinspired adaptive compliant control of a robot gecko applied for a space station. Based on the discussion above, the following conclusions can be drawn. The control strategy can effectively control the robot gecko to attach reliably and detach compliantly with force sensors through proper calibration. By employing different desired forces and impedance parameters for the front and hind legs, the robot gecko demonstrates remarkable disturbance rejection and adaptability to the external environment. The experiments demonstrated that the A-FS gait has stronger stability than TG and DG. Comparing to the existing robot used in the space microgravity environment, our robot gecko has three advantages: first, it has pneumatic flexible active attachment–detachment feet that can be used for facades, planes, curved surfaces, etc., through appropriate control. The biomimetic feet can effectively avoid stress concentration without damaging the special coating on the surface of the spacecraft. Second, it has a type of stable A-FS gait, making it more reliable in spatial applications. Third, it has achieved free-impact detachment and constant force tracking attachment, and the average tracking error is less than 1.64 N. Additionally, the presented control strategy is developed for our robot gecko, though it can also be used for other legged robots, such as the quadrupedal robot dog, with some modifications in the adaptative compliant control part. In the future, several issues shall be further investigated such as target tracking or three-dimensional obstacle avoidance. In particular, we plan to add a binocular vision system so that the robot gecko can achieve dynamic obstacle avoidance autonomously.

## Figures and Tables

**Table 1:** The parameters for motion control phase.

Description	Mark	TG		DG		A-FS		C-FS	
		Front Legs	Hind Legs	Front Legs	Hind Legs	Front Legs	Hind Legs	Front Legs	Hind Legs
stride distance	*S*_robot_ (mm)	90	90	90	90	45	90	45	90
stride height	*H*_robot_ (mm)	27	27	27	27	27	9	27	9
gait period	*T*_onegait_ (s)	16		6		32		32	
foot inflation time	*T*_in_ (s)	0.3		0.3		0.3		0.3	
foot deflation time	*T*_out_ (s)	0.5		0.5		0.5		0.5	

**Table 2:** The MPD and MRT of position tracking with different stiffness.

Stiffness Value	K = 0	K = 30	K = 60	K = 90
MPD (mm)	Not track	22.27	13.53	7.93
MRT (s)	Not track	3.57	1.86	0.89

**Table 3:** The MPD and MRT of position tracking with different damping.

Damping Value	B = 10	B = 30	B = 50	B = 70
MPD (mm)	13.19	10.19	13.17	12.16
MRT (s)	1.01	2.07	3.65	4.03

**Table 4:** The statistical results when desired attachment forces are 7 N–12 N.

Desired Attachment Force (N)	*F_d_* = 7	*F_d_* = 8	*F_d_* = 9	*F_d_* = 10	*F_d_* = 11	*F_d_* = 12
Mean preloading depth (mm)	4.64	5.21	6.67	8.23	9.45	10.36
Mean response time (s)	1.97	2.04	2.14	2.17	2.21	2.23
Mean force tracking deviation (N)	1.12	1.31	1.64	1.53	1.41	1.62

**Table 5:** The force control parameters of the triangular gait and diagonal gait.

Leg	*F_d_*	{*M*,*B*,*K*} of SWS	{*M*,*B*,*K*} of AS	{*M*,*B*,*K*} of SUS	{*M*,*B*,*K*} of DS
LF	8.0	{1,16,20}	{1,20,50}	{1,20,50}	{1,30,40}
RF	8.0	{1,16,20}	{1,20,50}	{1,20,50}	{1,30,40}
LH	8.0	{1,16,20}	{1,20,50}	{1,20,50}	{1,30,40}
RH	8.0	{1,16,20}	{1,20,50}	{1,20,50}	{1,30,40}

**Table 6:** The force control parameters of the alternate and continuous fulcrum-swing gait.

Leg	*F_d_*	{*M*,*B*,*K*} of SWS	{*M*,*B*,*K*} of AS	{*M*,*B*,*K*} of SUS	{*M*,*B*,*K*} of DS
LF	8.0	{1,16,60}	{1,20,50}	{1,20,50}	{1,30,40}
RF	8.0	{1,16,60}	{1,20,50}	{1,20,50}	{1,30,40}
LH	12.0	{1,16,60}	{1,16,55}	{1,16,55}	{1,25,45}
RH	10.0	{1,16,60}	{1,16,55}	{1,16,55}	{1,25,45}

## Data Availability

The data generated and/or analyzed during the current study are not publicly available for legal/ethical reasons but are available from the corresponding author upon reasonable request.
